# Beneficial Impact of Pork Dry-Cured Ham Consumption on Blood Pressure and Cardiometabolic Markers in Individuals with Cardiovascular Risk

**DOI:** 10.3390/nu14020298

**Published:** 2022-01-11

**Authors:** Silvia Montoro-García, Ángeles Velasco-Soria, Leticia Mora, Carmen Carazo-Díaz, David Prieto-Merino, Antonio Avellaneda, Domingo Miranzo, Teresa Casas-Pina, Fidel Toldrá, José Abellán-Alemán

**Affiliations:** 1 Department for Cardiovascular Risk, Faculty of Health Sciences, UCAM Catholic University of Murcia, Campus los Jerónimos, 30107 Murcia, Spain; velasco.angeles@gmail.com (Á.V.-S.); jabellan@ucam.edu (J.A.-A.); 2 Instituto de Agroquímica y Tecnología de Alimentos, CSIC, Av Agustín Escardino 7, 46980 Paterna, Valencia, Spain; lemoso@iata.csic.es (L.M.); ftoldra@iata.csic.es (F.T.); 3 Cátedra de Estadística “Big data”, UCAM Catholic University of Murcia, Campus los Jerónimos, 30107 Murcia, Spain; CCarazo@ucam.edu (C.C.-D.); dprieto@ucam.edu (D.P.-M.); 4 R&D Department, ElPozo Alimentación S.A., 30840 Alhama de Murcia, Spain; antonio.avellanedagoicuria@elpozo.com (A.A.); domingo.miranzo@elpozo.com (D.M.); 5 Clinical Analysis Service, University Hospital Virgen de la Arrixaca, Carretera Madrid-Cartagena, s/n, 30120 Murcia, Spain; tcasaspina@gmail.com

**Keywords:** pork dry-cured ham, hypertension, bioactive peptides, cardiovascular risk, blood pressure, hypercholesterolemia, ghrelin

## Abstract

Background: Evidence suggests that bioactive peptides reduce hypertension and affect certain metabolic pathways. Methods: Fifty-four volunteers with stage 1 prehypertension and/or hypercholesterolemia and/or basal glucose >100 mg/dL were recruited and randomized to pork dry-cured ham (*n* = 35) or cooked ham (placebo group; *n* = 19) for 28 days. After a wash-out period, meat products were changed for 28 additional days. Bioactive peptides composition and enzyme inhibitory activities of both products were characterized. Treatment comparisons for the main effects were made using a two (treatment) × two (times) repeated measures minus the effect of cooked ham (placebo). Results: 24 h mean systolic and diastolic pressures decreased up to 2.4 mmHg in the dry-cured ham period (treatment effect, *p* = 0.0382 y *p* = 0.0233, respectively) as well as the number of systolic pressure measures > 135 mmHg (treatment effect, *p* = 0.0070). Total cholesterol levels also decreased significantly after dry-cured ham intake (*p* = 0.049). No significant differences were observed between the two treatments for basal glucose, HOMA-IR index and insulin levels (*p* > 0.05). However, a significant rise of ghrelin levels was observed (treatment effect, *p* = 0.0350), while leptin plasma values slightly decreased (treatment effect, *p* = 0.0628). Conclusions: This study suggested the beneficial effects of regular dry-cured ham consumption on the improvement of systolic/diastolic blood pressures and facilitated the maintenance of metabolic pathways, which may be beneficial in the primary prevention of cardiovascular disease.

## 1. Introduction

Dietary strategies are recognized as the front line to prevent hypertension (HT), obesity and metabolic disorders [1,2]. In fact, HT is the most important condition for premature cardiovascular (CV) disease. In general, the restriction of consuming red/processed meat, eggs, fatty acids, and alcohol is recommended in a primary prevention setting [3]. Nonetheless, much effort has also been placed in the usefulness of bioactive molecules to support a health benefit, which, when effective, may provide a significant reduction in blood pressure (BP), cholesterol and basal glucose levels, among other metabolic effects [4,5,6]. Accumulating evidence further supports that other key biological pathways are involved for such bioactive compounds including altered serum lipid concentrations, inflammation, oxidative stress, insulin resistance and endothelial dysfunction [7]. The scientific community insists that these mechanisms of action need further investigation with original in vitro and in vivo approaches.

Bioactive peptides with angiotensin-converting enzyme (ACE; EC 3.4.15.1) inhibitory activity such as lacto-tripeptides have been shown to efficiently decrease BP. The formulation of these peptides into foods supports physiological functions in more than just nutrition. Pork dry-cured ham is a traditional Spanish food and one of the most appreciated meat products worldwide due to its high hedonic quality. Occasional consumption is recommended due to its moderate salt content despite the fact that it provides a varied amount of important nutrients and other bioactive compounds of interest [8]. The in vitro inhibitory capacities of bioactive peptides of this product have been previously reported for distinct enzymes and have been further tested in human cells and in mice by our groups [9,10,11,12]. Further, in a previous clinical study, our group already investigated the effects of consumption of dry-cured ham in healthy adults with normal-high systolic BP. The findings of this preliminary clinical trial illustrated for the first time that far from being a restricted food, the regular consumption of Spanish dry-cured ham impairs the evolvement of hypercholesterolemia and dysglycemia [13,14].

In this context, we have now conducted a second placebo-controlled clinical trial to evaluate how a modified dry-cured ham with an improved bioactive peptide composition could ameliorate medium-term BP control together with traditional CV risk factors in individuals with high CV-risk. Furthermore, this paper ranges from laboratory-based to clinical evidence and suggests that a specific dry-cured ham may improve cardiometabolic health through a myriad of mechanisms, underlying the specific bioactive peptides effects. 

## 2. Materials and Methods

### 2.1. Study Population

Participants were recruited from distinct primary attention units of the Región de Murcia (Spain) and among staff of the Catholic University of Murcia (UCAM). 

Inclusion criteria were the following: aged 20–65 years; had a 130 ≤ systolic BP ≤ 150 mmHg; diastolic BP > 80 mmHg (home BP, average of 3 readings taken after 5 min rest), body mass index (BMI): 20.0–35.0 kg/m^2^; basal cholesterol level >200 mg/dL and/or basal glucose level >100 mg/dL, informed consent. Main exclusion criteria were similar to our previous study [13]. 

The study protocol was approved by the Clinical Research Ethics Committee of the *Servicio Murciano de Salud* (Area 1, Región de Murcia, Spain), the UCAM Ethics Committee, and was conducted in accordance with the Declaration of Helsinki. This trial was registered in November 2021 at ClinicalTrials.gov: Identifier CE111703. 

### 2.2. Study Design

This study was a two-arm randomized, placebo-controlled cross-over trial (RCT) with two four-week periods separated by a two-week washout, assessed from January 2020 to December 2020. From a total of 60 individuals who were recruited, fifty-four patients with CV risk factors and untreated normal-high BP (65% male) participated in the study. None of the withdrawals were due to the meat products. A washout period of two weeks was included for all the participants before entering the clinical trial to assure they refrained from restricted foods (plant sterols, other cured meats) and drugs (in any). One group (*n* = 35) received a controlled salt dry-cured ham of >12 months proteolysis (interventional product), while the other (*n* = 19) received cooked ham (placebo-control product), each for 28 days. After a two-week wash out period, the groups exchanged roles for another 28 days. In this way, each group had consumed both meat products for 28 days. The duration of the washout was in line with other peptide dietary intervention studies [13,15]. Participants received meat products weekly in individual daily sachets: interventional (dry-cured ham, 80 g/day) or placebo-control (cooked ham, 100 g/day) products. The study was double blind and the participants which of the hams was supposed to be the interventional product.

Enrolled participants were randomly assigned to their treatment by only one investigator. The study arms were similar for distinct parameters (age, BMI, gender etc.). The primary outcomes were systolic/diastolic BP day and night mean; and the secondary outcomes included fasting plasma lipids/glucose levels and changes in plasmatic biomarkers of the CV status.

### 2.3. Pork Meat Products

Spanish dry-cured hams and placebo cooked hams under investigation were manufactured specifically for the current study by a local Spanish company (*ElPozo Alimentación*, *S.A*., Murcia, Spain). Dry-cured hams were made according to traditional procedures with several modifications as follows. Raw hams from six months old pigs belonging to the genetic crossing (Landrace-Large White × Duroc) standardized by the company were used. Hams were chilled until reading an internal temperature below 3 °C before proceeding to the salting and subsequent curing process. A singular salting and curing process (*ElPozo Alimentación*, S.A.) was followed to control the exact amount of salt in each piece and to favor proteolytic processes during the maturation of the hams. A first stage consisted of forced and controlled cold salting in a single stage using a mixture of sea salt and nitrifying salt, followed by a stabilization stage at a temperature below 4 °C and 75–85% relative humidity for 60 days. This was followed by a ripening period (12–30 °C, with relative humidity progressively reduced to 65%) for eight months and, finally, an aging period (18–22 °C, relative humidity 70–80%) of two months. The total length of the dry-curing process was 12–14 months depending on the cured-ham size. The muscle in the final dry-cured hams had pH values within the range of 5.9 to 6.2, the water content of 48 to 52%, and the salt content of 3.5 to 4.0%. Superior category cooked hams supplemented with extra salt content were used as a placebo meat product with a 2.5% salt content (without active peptides). For the present clinical study, both meat products were presented in slices format within modified atmosphere packaged and labelled with safety information (consumption limit date, nutritional composition, and best storage condition), as if they are for commercial purposes but without the Brand. The composition of the products is depicted in Table 1. Individuals were asked to eat a packet throughout the day but without cooking the meat products.

### 2.4. Identification of Bioactive Peptides in Hams by Mass Spectrometry in Tandem

Liquid chromatography and tandem mass spectrometry (LC–MS/MS) were used in the identification following the previous published methodology [16].

ProteinPilot v 4.5 default parameters were used to generate peak lists directly from 5600 TripleTof wiff files. The Paragon algorithm of ProteinPilot was used to search: Uniprot Mammals 03 2018) 2,753,628 proteins searched. All databases were searched with the following parameters: no enzyme specificity and no Cys. The protein grouping was done by the Pro Group algorithm, so a protein group in a Pro Group Report is a set of proteins that share some physical evidence.

Unlike sequence alignment analyses where full length theoretical sequences are compared, the formation of protein groups in the Pro Group is guided entirely by observed peptides only. Since the observed peptides are determined from experimentally acquired spectra, the grouping can be guided by usage of spectra. Then, unobserved regions of protein sequence play no role in explaining the data. Proteins showing an unused score >1.3 were identified with confidence ≥ 96% according to the following equation.

ProtScore= −log ((1 − (% confidence)/100))

Percent confidence of 95% was used in the identification.

### 2.5. In Vitro Activity of Bioactive Peptides

The in vitro antihypertensive activity measured as the inhibitory activity of the ACE was assayed in peptide extracts obtained from both meat products. Moreover, the inhibitory activity over the enzyme hydroxymethylglutaryl-coenzyme A reductase (HMG-CoAR) and the binding capacity of bile acids were also tested for the same extracts as a measure of hypocholesterolemic activity.

#### 2.5.1. ACE Inhibitory Activity Assay

The ACE inhibitory activity was measured according to the method developed previously by our group [17]. 

#### 2.5.2. HMG-CoAR Inhibitory Activity Assay

The assay was done using the commercial kit from Sigma-Aldrich (HMG-CoA Reductase Assay Kit (Catalog number: CS1090; Sigma-Aldrich, St. Louis, MO, USA). The protocol was carried out according to the manufacturer’s instructions. The absorbance was read at 340 nm in a CLARIOstar microplate reader (BMG LABTECH, Ortenberg, Germany), from 0 to 10 min. The HMG-CoAR dependent oxidation of NADPH in the absence and presence of inhibitors was measured. Then, enzyme inhibition was calculated as follows:

% HMG-CoAR Inhibition = ((∆Abs 100% activity − ∆Abs sample))/(∆Abs 100% activity) × 100

%Inhibition = ((ΔAbs 100%activity − ΔAbs Sample)/ΔAbs 100%activity) × 100

where ΔAbs indicates the difference between absorbance values after 10 and initial time.

#### 2.5.3. Peptide Extracts Binding Capacity of Bile Acids 

The bile acids binding capacity of the dry-cured ham extract was analyzed by reverse phase HPLC using a Luna5 C18 column (250 × 4.60 mm) from Phenomenex [18]. Glycocholic acid was used as standard to control the binding capacity of the extract.

### 2.6. Characteristics and Dietary Habits of Participants

Four visits were performed by a qualified nurse and included standardized questionnaires updating of personal anamnesis and a physical examination with anthropometric data collection with bioelectrical impedance using a Tanita BC-541 (Illinois, USA) (weight, height, BMI, body/visceral fat percentage, and muscle). The impedance procedure took approximately 60 s. A list of restricted foods (plant sterols, cured meat, chips, pickles, dry soups) was given at the beginning of the study to avoid the excessive consumption of salt and other cured meat products. Nutritional interviews with a food frequency questionnaire (FFQ) to collect dietary recall (at least a three-day recall) were performed to estimate nutrients and salt intake before and after meat products and to ensure that the diet had not changed. The ham intake and food restriction were monitored in a weekly visit to assess compliance to the study. Participants were asked to bring the remaining sachets with them.

### 2.7. Blood Pressure Monitoring

When the mean of home readings satisfied systolic and diastolic BPs above 130 and 80 mmHg, respectively, individuals were recruited. During the clinical study, a 24 h ambulatory BP monitoring (ABPM) was assessed for all the participants at four different time-points (before/after interventional and control products). One trained research nurse placed the ABPM and gave instructions to the patients about how to act, work and sleep. Volunteers were appointed for a blood extraction and 24 h holter using a digital non-invasive automatic manometer device OMRON M24/7 BP5. Readings were automatically obtained at 20- and 30-min intervals during daytime and nighttime, respectively. Readings with <65% measurements were rejected. Separate systolic BP (SBP) and diastolic BP (DBP) averages were taken into consideration for 24 h, day- and night-values. Ambulatory mean pulse pressure (PP) was calculated as PP = SBP − DBP. Mean arterial pressure (MAP), which measures the resistance of arteries, was estimated by: MAP = DBP + 1/3(PP) [19].

### 2.8. Blood Sampling and Biochemical Determinations

Blood was taken before and after each period (interventional/control ham), after 8 h of fasting followed by a two week wash out period (for a total of four time-points). 

The fasting blood lipid profile (total cholesterol, high-density lipoprotein-cholesterol [HDL], low-density lipoprotein-cholesterol [LDL] and triglycerides [TG]); hemogram, glucose, Na^+^, K^+^, transaminases and creatinine levels were analyzed. Insulin resistance was estimated by the homeostatic model assessment for insulin resistance (HOMA-IR) index [20]. Glycated hemoglobin (HbA1c) was measured using ion-exchange high performance liquid chromatography. Urine was also collected for 24 hours and daily urinary sodium excretion was measured using the ion selecting electrode method [21].

### 2.9. Plasma Biomarkers

Plasma samples (citrated and EDTA blood) were used to quantify levels of soluble markers of the CV pathophysiological status: inflammation (interleukin-6 [IL-6] and C reactive protein [PCR]), endothelial dysfunction (von Willebrand factor [vWF] and plasminogen activator inhibitor-1 [PAI-1]), metabolic/appetite (leptin, ghrelin and adiponectin) and oxidative status (oxidative LDL [oxLDL]). Samples were stored at −80 °C until assayed.

Measurement of vWF (vWF:Ag) was performed by using enzyme-linked immunosorbent assay (ELISA), using the kit (Technoclone Cat. No. 5450201), according to the manufacturer’s recommendations (interassay CV < 5%). The levels of oxLDL were also measured by an ELISA kit (FineTest Cat. No, EH0943, Wuhan, China). The levels of plasma oxLDL were expressed in units (U/mL). The coefficients of variation of intra-assay and inter-assay were 4.1 and 6.7%, respectively.

Using magnetic bead-based assays, the plasma adiponectin, leptin, ghrelin, P-selectin, PAI-1, IL-6, and PCR concentrations were measured. Two customized Procartaplex multiplex kits (Thermo Fisher Scientific) were used following the manufacturer’s instructions; they utilized polystyrene bead-based technology to measure the seven markers (adiponectin and PCR in one kit and the rest of the markers in the other customize kit) [22]. Based on the measurements of standard concentrations provided by the manufacturer, standard curves were utilized to convert optical density values into concentrations (pg/mL). Supernatant samples were thawed once and clarified by centrifugation at 10,000× *g* for 10 min. Next, the plate was loaded into the Luminex MAGPIX^®^ (Merck Millipore) system for reading. On both kits, the intraassay was calculated based on duplicated samples for all the biomarkers (<10%). The inter-assay CV ranges for this method were 5.2% for P-selectin, 5.6% for ghrelin, 6.0% for PAI-1 and leptin, 6.5% for IL-6, 6.9 % for PCR, and 7.1% for adiponectin.

The searchlight platform had acceptable intra-assay variability (intra-assay coefficient of variation (CV%) range for all analytes of 9.1–13.7), but unacceptably high inter-assay variability (CV% range for all analytes 16.7–119.3) suggesting plate-to plate variability. Similar assays for individual cytokines on the R&D platform had an intra-assay CV% range of 1.6–6.4 and an inter-assay CV% range of 3.8–7.1.

### 2.10. Sample Calculation and Statistical Analyses

With a sample size of 50 individuals, a power greater than 80% was calculated to detect a difference of effect of three units of PAS between the two types of meat products, assuming a deviation of five points in this measure between individuals and a type error- I 5%. Data are expressed as mean ± standard deviation (SD) and confidence interval [CI 95%] for normally distributed data. The present analyses are focused on estimating differences at different time points by the linear random effect model assuming a different “baseline” value for each subject using the program R v3.2.4 and the lme4 package. A *p*-value of <0.05 was considered statistically significant. SPSS 21.0 software was used for the rest of statistical analyses (SPSS, Inc, Chicago, IL, USA).

## 3. Results

### 3.1. Meat Products Characterization

The macronutrients composition of the tested meat products is shown in Table 1. Protein and fats percentages were higher in dry-cured ham. It is important to mention that the salt content of the tested dry-cured ham (3.5%) was lower than conventional dry-cured products and was more similar to the salt content of the tested cooked ham (2.5%) used as a placebo-control. Besides, cooked ham did not contain relevant amounts of free amino acids, and the studied biologically active peptide sequences were not present (Table 1).

### 3.2. In Vitro Inhibitory Bioactivies

Traditionally, the ACE inhibition activity has been used as the main in vitro method for antihypertensive activity of bioactive peptides. On average, dry-cured ham was shown to have higher ACE inhibitory activity than cooked ham (Table 2) (*p* < 0.05). The results of the inhibition of HMG-CoAR inhibitory activity are presented in Table 2, and a difference higher than cooked ham was observed (*p* < 0.05).

**Figure 1:**
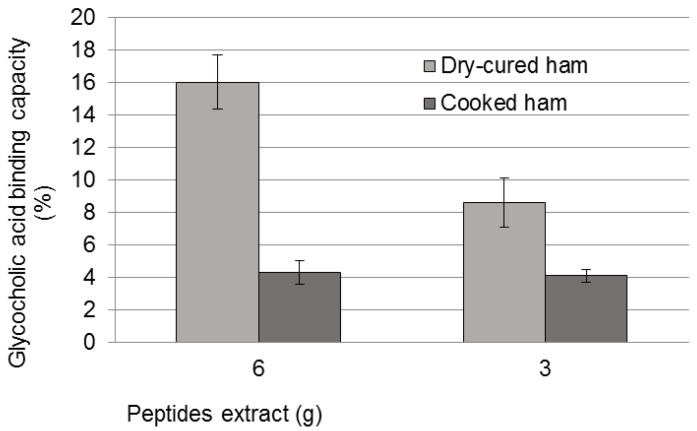
Sequestering capacity of bile acids measured in glycocholic acid presence for the two meat products.

Another in vitro method consisted of the measurement of bioactive peptides binding capacity of bile acids. The results indicated a sequestering capacity of 16% glycocholic acid for an amount of peptide extract equivalent to 6 g of dry-cured ham, while the cooked ham displayed only 4% of sequestering capacity (*p* < 0.05, Figure 1). Interestingly, a positive dose-dependent relation for the dry-cured peptide extract was also found.

### 3.3. Baseline Characteristics of the Study Population

Fifty-four individuals (49.00 ± 10.28 years old; 65% males) successfully completed the study. Readings of systolic BP were above 130 mmHg when measured with home BP monitoring (data not shown), considered as stage 1 hypertension or pre-hypertension by major clinical guidelines [23]. Table 3 shows the data of SBP and DBP measured with 24-h ABPM. They individuals were generally middle-aged, and thirteen of them (24.07%) were obese, defined as BMI >30 kg/m^2^. On average, the group was hypercholesterolemic (212.98 ± 37.48 mg/dL), with above optimal TG and LDL levels (118.53 ± 62.43 and 131.94 ± 30.35 mg/dL, respectively) (Table 3) [24]. Thirty percent had fasting glucose > 100 mg/dL, and among them, three had highly impaired fasting glucose (>130 mg/dL), and two patients were confirmed as non-treated diabetics (>200 mg/dL). Hemoglobin A1c (HbA1c) (glycated haemoglobin) was 5.75 ± 0.64%, which confirms the increased relative CV risk of this population [25]. Thus, the patients presented borderline-high BP levels together with borderline cholesterol or glucose, which further increases the global CV risk.

At baseline, subjects were randomly allocated into two groups and well matched for all the considered clinical characteristics, including sex and age (all *p* > 0.05) (Table 3). Similar dietary habits from the randomization (Table 3) until the end of the study were confirmed by FFQ; individuals did not change their dietary habits during the study (Table A1). 

**Figure 2:**
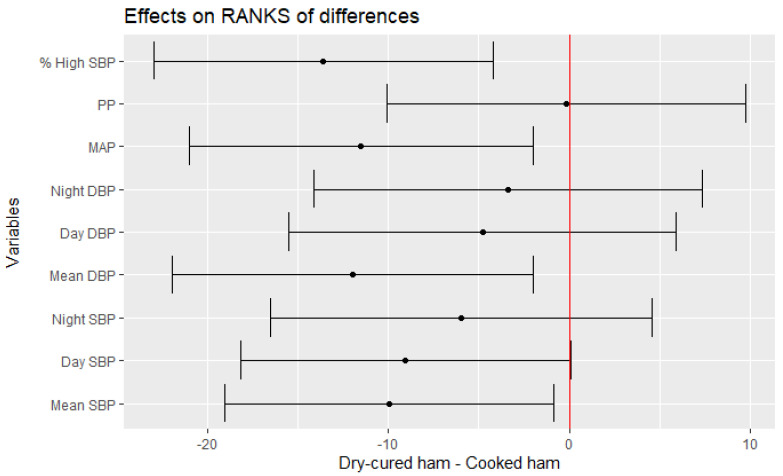
Estimate of the mean range of the effect of dry-cured ham intake minus placebo-controlled product and its 95% confidence intervals for blood pressure values.

The effect of dry-cured ham consumption was calculated considering the effect of the placebo-controlled product (cooked ham) as well. Thus, the following statistical analyses calculate the effect of the treatment in the distinct parameters after resting the effect of the placebo-control product by linear random effect model assuming a different “baseline” value for each subject (Table 4). Figure 2 shows that participants after interventional intake experienced a significant improvement in the mean changes of Mean 24 h-SBP (−2.41 mmHg vs. baseline), Day SBP (−2.05 mmHg vs. baseline) and Mean 24 h-DBP (−2.49 mmHg vs. baseline). Moreover, reductions of MAP and % SBP > normal [% High SBP] were also found significant (*p* = 0.0222 and *p* = 0.0070, respectively) after the interventional treatment (Table 4, Figure 2).

**Figure 3:**
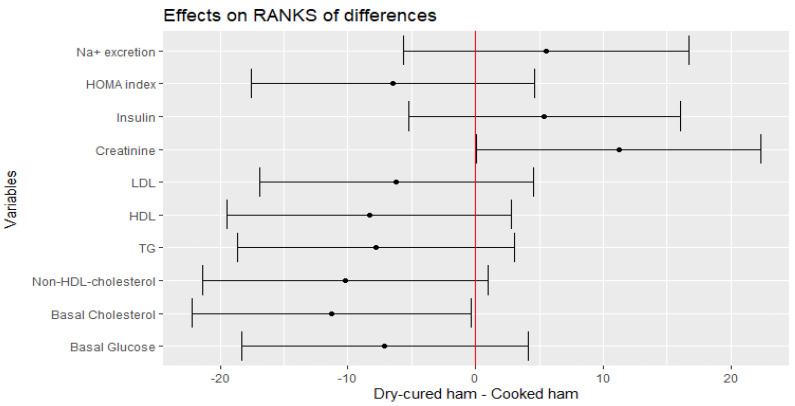
Estimate of the mean range of the effect of dry-cured ham intake minus placebo product and its 95% confidence intervals for hematological and clinical parameters.

The mean changes in fasting glucose, non-HDL-cholesterol, LDL, HDL and TG levels after the intake of the treatment meat product remained unchanged (Figure 3). However, there was an effect of treatment for total cholesterol for ranks of differences (*p* = 0.0487) (Figure 3), which is translated in an estimate change of −9.7467 (CI −20.7100, 1.2166) mg/dL. Moreover, when TG was log-transformed due to high variation, a negative trend was envisaged (−10.3171, CI −21.2649, 0.6307; *p* = 0.071). The HOMA-IR index and the 24 h-Na^+^ excretion did not change either after the interventional intake (Figure 3).

### 3.4. Plasmatic Biomarkers of the CV Pathophysiological Status

The clinical use of biomarkers can lead to modest improvements in the evaluation of the CV status beyond the above traditional CV risk factors [26]. 

**Figure 4:**
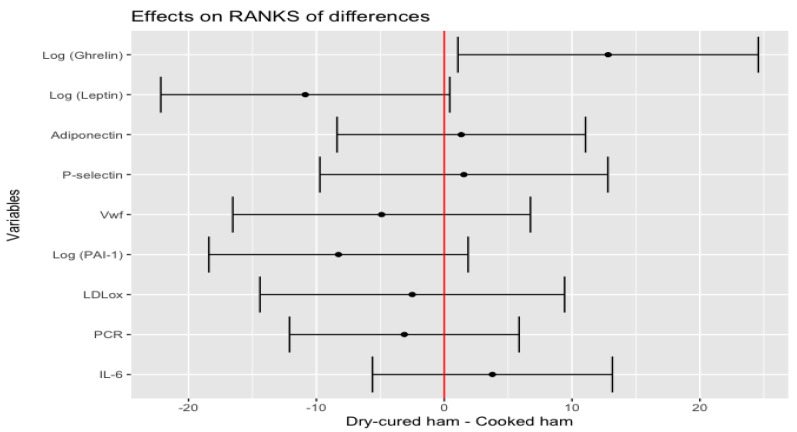
Estimate of the mean range of the effect of the dry-cured ham intake minus the placebo-control product and its 95% confidence intervals for biomarkers of the CV pathophysiological status.

Linear regression and Pearson correlation found that plasma levels of endogenous adipocyte-derived and food intake mediators, such as adiponectin and ghrelin, associated with basal glucose (R^2^ = 19%, *p* < 0.001 and R^2^ = 8%, *p* < 0.006, respectively) at baseline status (Figures S1 and S2). Additional analysis of plasma biomarkers revealed the best linear curve fitting for adiponectin, leptin, ghrelin, PCR, PAI-1 and P-selectin by logarithmic transformation (data not shown). Increased plasma levels of log-transformed ghrelin and reduced of log-transformed leptin, were associated with dry-cured ham intake minus placebo-control product effect (*p* = 0.0350 and *p* = 0.0628, respectively) (Figure 4). 

## 4. Discussion

Bioactive compounds, either in the form of crude extracts or in foods, might regulate the multifactorial atherosclerotic process. Particularly, bioactive peptides receive growing attention due to its pleotropic actions in the organism, such as participation in the glucose and fat metabolism, modulation of appetite, the dilation of blood vessels as well as inhibiting inflammation or apoptosis [27]. The purpose of this study was to prove the beneficial effects of a particular type of dry-cured ham with in vitro characterized bioactive peptides on hemodynamics, clinical parameters and biomarkers of the cardiometabolic health in humans with an incipient pathological CV status. Indeed, daily consumption of 80 g of the interventional product reduced 24 h-systolic/diastolic BP in >2.4 mmHg, and total cholesterol in −9.74 mg/dL. Indeed, day BP values were more affected than night BP values, perhaps because the short peptides enter the blood circulation shortly after oral ingestion (3–4 h), which occurs during the day [28]. Other blood biomarkers (inflammation or oxidation) and hemodynamics remained unaltered after the consumption of this cured ham. Besides, this study includes an initial run-in period, which is different from our previous clinical study [13].

### 4.1. In Vitro Biological Activities

According to WHO, the first strategy in the prevention and management of CV disease should use dietary approaches with the consumption of bioactive compounds when available [29,30,31]. Dry-cured meats constitute very good sources of several nutrients and bioactive peptides [8,32]. Firstly, the in vitro approaches used for this study highlighted the ACE and HMG-CoAR inhibitory activities of the interventional product, which are in accordance with earlier observations in other meat products [33]. Given the multifunctional activities of the bioactive peptides, the binding capacity for bile acids of a peptide extract was furthermore evaluated in order to support a potential synergistic reduction of total cholesterol [34]. Herein, the bioactive peptides from the interventional product could exert broad mechanisms of action to be tested in vivo, and in a CV pathological context. Our previous exciting findings with healthy volunteers suggested a role of bioactive peptides from dry-cured ham in reducing cholesterol, LDL and basal glucose levels [13]. It encouraged us to compare the effect of such bioactive peptides in distinct physiopathological settings. Thus, the present data provide additional evidence because they were focused on individuals with CV risk such as high BP, hypercholesterolemia and/or high glucose level. 

### 4.2. Key Metabolic Pathways

In all HTA guidelines, patients are recommended not to consume cured meats due to its high salt content, which would lead to water retention and consequently to a volume-dependent increased BP [35,36]. However, the current data support the position that although the above reasoning is feasible, dry-cured ham intake does not compromise the BP in healthy volunteers nor in individuals with prehypertension (stage 1) [13,37], since Na+ excretion remained stable. Therefore, the supposed pressor effect due to the entry of extra sodium after ingestion could be counteracted by the action of these bioactive peptides. Moreover, the regular intake of this particular dry-cured ham did not disturb the metabolic profile of the CV patient; in fact, a trend to decrease the glycemic profile and improve the lipid profile was found. The actions that bioactive peptides play on the glycemic profile are currently under investigation [38], and a possible dipeptidyl peptidase-IV and α-glucosidase inhibition or at the level of glucose intake had been suggested [39,40,41]. Regarding the lipid metabolism, the consumption of dry-cured ham could interfere at the digestive level with the absorption of bile salts rich in cholesterol, acting as a sequestrant of bile salts, an action like that exerted by resins [42]. In fact, experiments carried out here demonstrate the in vitro capacities of such peptides. Intervention with dry-cured ham in participants at high risk for CV disease improves the elevated levels of lipids such as basal cholesterol (*p* = 0.048), although other variables remained unchanged: non-HDL-cholesterol (*p* = 0.08020) and log TG (*p* = 0.0712).

### 4.3. Adipokines and Inflammation

Ghrelin is a gastric peptide with CV actions, and increasing evidence demonstrates that exogenous administration of ghrelin closely inhibits proatherogenic changes in experimental models [43,44,45]. Further research regarding the role of the CV protective effect of ghrelin would be of great help, including the lowering of BP, the regulation of atherosclerosis, and insulin resistance [46,47,48]. An interesting study recently reported ghrelin-stimulating bioactive peptides derived from casein with potent effects in rats and in vitro [49]. In accordance with our clinical data, another animal study also revealed a decrease in BP after ghrelin administration [50]. Deciphering the specific molecular mechanism(s) of bioactive peptides is far from the scope of the current study, thus, whether BP decreases because of the increased ghrelin levels or by ACE inhibition remains to be investigated. Nonetheless, to our knowledge, this is the first human clinical study that illustrates the positive increase of ghrelin levels after bioactive peptide consumption in humans. The current literature provides further evidence that ghrelin modulates insulin secretion which influences glucose homeostasis downstream as well [48]. Contrary to our expectations, the data did not find a significant reduction of the basal glucose, HOMA-IR nor insulin levels after the interventional treatment. This might be due to the complexity of the cardiometabolic processes, as some of the effects of ghrelin are still debated in the literature [51].

On the other hand, leptin and adiponectin are two adipocyte-derived hormones which regulate food intake and energy expenditure [52]. Individuals with severe coronary artery disease, metabolic syndrome or abdominal obesity display decreased adiponectin and increased leptin plasma levels [53]. The results of this study showed a trend in the reduction of leptin levels (*p* = 0.068) which, once more, supports the idea of a positive regulation of metabolic functions for bioactive peptides. Leptin further links inflammation —by upregulating proinflammatory cytokines—and insulin resistance [54]. Despite the potential anti-inflammatory role of bioactive peptides, levels of IL-6 and PCR remained unaltered in the current clinical study. Quite often, the prevention of CV disease requires the modulation of the inflammation that accompanies cardiovascular risk factors such as hypercholesterolemia or *diabetes* [55,56]. Nonetheless, the inflammatory response appears with small danger stimuli and thus it is somehow linked to any dysfunctional process. In the current study, the hemodynamic and metabolic status of the population ameliorate after the treatment, but it was still insufficient to modulate chronic inflammation. Despite the fact that adipokines are not routinely used as clinical biomarkers, changes in such circulating levels are reflective of the cardiometabolic responses, which can be used to test the efficacy of the dry-cured ham consumption. 

## 5. Conclusions

Bioactive peptides naturally present in the assayed dry-cured ham positively affect plasmatic levels of molecules involved in the hypertensive and metabolic processes. Due to its apparent benefit in combating hypertension, hypercholesterolemia and *diabetes*, these food-derived peptides are attractive to the food industry and consumers because of their potential for use in dietary management. Besides, this study also increases the current evidence on the complex regulatory mechanisms exerted by these peptides by combining in vitro approaches and plasmatic biomarkers. 

## 6. Limitations

Limited translatability of the treatment effect might be due to the high heterogeneity of the study population (BMI and fat content were not completely randomized). Unfortunately, patients with higher CV risk (higher BP, cholesterol, or glucose levels) could not be recruited because of the requirement of medication. Furthermore, the researchers were not blinded, which may negatively affect the final outcomes. Likewise, it is possible that effectors other than peptides or lipids could have been released during the processes of proteolysis and lipolysis of dry-cured ham. Finally, conducting the quantification of plasma circulating bioactive peptides could have confirmed their presence, but this is still a challenge. 

## Figures and Tables

**Table 1:** Nutritional composition and total content of active peptides of interventional and placebo-control meat products.

	Dry-Cured Ham	Cooked Ham
Energy value (Kcal/100 g)	241.5 ± 9.3	97.7 ± 3.9
Fats (%)	12.1 ± 2.2	2.5 ± 0.3
Saturated Fats (%)	4.4 ± 0.3	0.9 ± 0.01
Monounsaturated Fats (%)	6.2 ± 0.3	1.3 ± 0.1
Polyunsaturated Fats (%)	1.5 ± 0.3	0.3 ± 0.02
Carbohydrates (%)	0.0	0.3 ± 0.08
Sugars (%)	0.0	0.3 ± 0.07
Proteins (%)	32.8 ± 2.0	18.5 ± 0.4
Salt (%)	3.5 ± 0.3	2.5 ± 0.2
Sodium (ppm)	13672 ± 1143	9850 ± 622
Total Nitrogen (%)	5.24 ± 0.5	2.97 ± 0.055
Soluble Nitrogen (%)	1.95 ± 0.07	0.45 ± 0.006
Denatured Nitrogen (%)	1.92 ± 0.03	0.44 ± 0.021
Non-Protein Nitrogen (%)	1.49 ± 0.02	0.46 ± 0.006
Free Amino acids (mg/100 g dm)	9656 ± 715	-
Proteolysis Index (%)	28.5	-
Previously identified biologically active peptides that are present in the sample	AAATP, PAPPK, KAAAAP, AMNPP, IKLPP, AAPLAP, KPVAAP, KPGRP, PSNPP, IAGRP, KVLPG, TGLKP, KAAAATP	None

**Table 2:** Inhibitory activities of interventional and placebo-control meat products.

Inhibitory Activity, %	Dry-Cured Ham	Cooked Ham	*p* Value
Angiotensin converting enzyme (ACE)	85.22 ± 2.4	50.12 ± 0.26	*p* < 0.05
HMG-CoAR	56.6 ± 3.1	45.9 ± 3.5	*p* < 0.05

ACE: Angiotensin-Converting Enzyme; HMG-CoAR: Hydroxymethylglutaryl-Coenzyme A Reductase.

**Table 3:** Clinical characteristics of participants with normal-high BP expressed as mean ± standard deviation.

	All (*n* = 54)	Cooked Ham First (*n* = 19)	Dry-Cured Ham First (*n* = 35)	*p*-Value
Age, years	49.00 ± 10.28	49.42 ± 11.98	49.05 ± 9.4	0.703
Gender (male)	65%	68%	63%	0.612
BMI, (kg/m2)	28.01 ± 4.79	25.56 ± 4.38	29.30 ± 4.34	0.065
Fat content, %	31.84 ± 9.12	27.88 ± 8.96	34.06 ± 8.55	0.069
Mean 24 h-Systolic BP, mmHg	127.00 ± 10.85	124.05 ± 9.32	128.38 ± 11.51	0.103
Mean 24 h-Diastolic BP, mmHg	74.12 ± 8.15	73.26 ± 7.93	75.24 ± 8.25	0.363
Basal Glucose mg/dL	98.57 ± 24.01	105.89 ± 36.22	93.96 ± 10.68	0.277
Basal Cholesterol, mg/dL	212.98 ± 37.48	191.85 ± 26.51	216.54 ± 42.40	0.195
TG, mg/dL	118.53 ± 62.43	102.84 ± 49.33	130.30 ± 68.47	0.105
HDL, mg/dL	58.18 ± 30.35	60.95 ± 14.29	56.79 ± 14.58	0.353
LDL, mg/dL	131.94 ± 30.35	122.74 ± 24.92	135.21 ± 34.19	0.201
non-HDL-Cholesterol, mg/dL	157.38 ± 39.32	143.68 ± 26.78	164.25 ± 42.00	0.071
Creatinine, mg/dL	0.86 ± 0.17	0.85 ± 0.16	0.86 ± 0.18	0.957
Insulin, mIU/L	11.92 ± 8.31	11.72 ± 10.80	11.96 ± 6.51	0.232
HbA1c, %	5.75 ± 0.64	5.98 ± 0.94	5.60 ± 0.30	0.104
**Food groups**				
Dairy (1–2/day), %	85.8	79.5	86.1	0.487
Fruit and vegetables (2–3/day), %	84.2	85.2	78.8	0.302
Cereals (2–3 day), %	42.5	37.2	44.6	0.239
Poultry (2–3/week), %	33.5	37.2	31.0	0.195
Red and other processed meat (3–4/week), %	76.5	73.7	77.1	0.587
Nut (3–4/week), %	25.0	25.3	24.7	0.955
Red wine, 4–7/week, %	8.5	8.9	8.4	0.927
Olive oil, daily, %	93.5	91.8	94.5	0.803

BMI: Body Mass Index; BP: Blood Pressure; TG: Triglycerides; HDL: High density lipoprotein; LDL: Low density lipoprotein; HbA1c: glycated hemoglobin. BP was measured with 24 h-ABPM (OSROM). T-test was performed.

**Table 4:** Modelled difference of the changes in blood pressure parameters when eating dry-cured ham minus when eating placebo-control product.

Variable	Cooked Ham	Dry-Cured Ham	Dry-Cured—Cooked Ham Effect	P-ori	*p*-Value
BMI, (kg/m2)	0.08 (0.54)	−0.09 (0.65)	−0.15 (−0.39 to 0.09)	0.21470	0.39180
Fat content, %	−0.07 (1.86)	−0.40 (1.82)	−0.22 (−0.89 to 0.45)	0.52420	0.89420
Mean 24 h-Systolic BP	0.65 (4.66)	−1.62 (6.89)	−2.41 (−4.39 to −0.43)	**0.02190**	**0.03820**
Day Systolic BP	0.06 (5.50)	−1.58 (7.54)	−2.05 (−4.33 to 0.23)	0.08630	**0.05830**
Night Systolic BP	2.00 (6.24)	0.02 (7.90)	−1.83 (−4.46 to 0.79)	0.17810	0.27250
Mean 24 h-Diastolic BP	1.08 (4.04)	−1.20 (5.35)	−2.49 (−4.14 to −0.84)	**0.00500**	**0.02330**
Day Diastolic BP	0.81 (5.92)	−1.06 (8.00)	−2.09 (−4.66 to 0.48)	0.11810	0.38610
Night Diastolic BP	0.57 (9.14)	−0.41 (12.29)	−2.20 (−6.37 to 1.97)	0.30650	0.53660
MAP	0.94 (3.82)	−1.34 (5.56)	−2.47 (−4.10 to −0.85)	**0.00480**	**0.02220**
% High SBP	2.61 (7.76)	−2.73 (8.67)	−3.91 (−6.76 to −1.07)	**0.01040**	**0.00700**

Significant values appears in bold. Cooked ham = Effect (difference after−before) of eating cooked ham on the variable, dry-cured ham = Effect (difference after−before) of eating dry-cured ham on the variable. Dry-cured−cooked ham effect = effect difference (dry-cured effect−cooked ham effect) modelled with adjustment for order of consumption of each type of ham and for baseline value of the variable centred on the mean. P-ori = *p*-value of the difference of effects on the original scale, P-ran = *p*-value of the difference of effects on the scale of ranges.

## Supplementary Materials

The following supporting information can be downloaded at: https://www.mdpi.com/article/10.3390/nu14020298/s1, Figure S1. Linear regression and Pearson correlation between basal glucose and adiponectin at baseline status. Figure S2. Linear regression and Pearson correlation between basal glucose and ghrelin at baseline status.

## Appendix A

When the intake of both meat products was considered but not the period of consumption (first or second treatment), individuals did not experience any change either in anthropo-metric and hematochemical parameters, except for HDL, which significantly decreased (3.93% versus baseline) after dry-cured ham intake (*p* < 0.05), and night SBP increased (1.38% versus baseline) after cooked ham consumption (Table A1). The FFQ estimated the food groups intake and allow to discard changes in the dietary habits of the population.

**Table A1:** Changes in anthropometric, hemodynamic, biochemical data and food groups intake from baseline to end of treatment, expressed as mean ± standard deviation.

	Baseline Dry-Cured Ham (*n* = 54)	End Dry-Cured ham	Baseline Cooked Ham (*n* = 54)	End Cooked Ham
BMI, (kg/m2)	27.97 ± 4.67	28.18 ± 4.56	27.93 ± 4.60	29.29 ± 4.30
Fat content, %	33.62 ± 7.53	33.11 ± 7.57	31.73 ± 8.92	31.10 ± 8.89
Mean 24 h-Systolic BP, mmHg	126.80 ± 10.97	124.28 ± 10.18	125.78 ± 10.54	126.08 ± 10.68
Day Systolic BP, mmHg	130.76 ± 10.92	128.54 ± 10.40	130.76 ± 11.08	130.58 ± 10.67
Night Systolic BP, mmHg	119.06 ± 12.60	117.51 ± 12.10	116.65 ± 11.55	118.26 ± 12.29 *
Mean 24 h-Diastolic BP, mmHg	76.67 ± 8.02	74.86 ± 7.58	76.02 ± 8.19	76.94 ± 8.24
Day Diastolic BP, mmHg	79.78 ± 9.69	78.31 ± 9.76	80.16 ± 8.74	80.96 ± 8.75
Night Diastolic BP, mmHg	70.12 ± 8.07	68.94 ± 13.18	70.27 ± 9.59	70.46 ± 9.18
MAP, mmHg	93.40 ± 8.34	89.52 ± 14.92	90.82 ± 15.17	93.4 ± 8.54
Basal Glucose, mg/dL	98.20 ± 27.70	93.40 ± 11.08	99.51 ± 29.36	98.15 ± 23.69
Basal Cholesterol, mg/dL	213.59 ± 40.38	209.69 ± 37.83	210.79 ± 35.77	211.81 ± 36.52
TG, mg/dL	119.83 ± 60.45	112.44 ± 57.31	136.38 ± 163.15	122.15 ± 61.79
HDL, mg/dL	57.79 ± 14.14	55.60 ± 13.08 *	56.58 ± 15.08	56.34 ± 13.93
LDL, mg/dL	130.79 ± 31.23	131.35 ± 32.43	130.79 ± 30.41	130.38 ± 30.46
Non-HDL-Cholesterol, mg/dL	158.88 ± 39.11	152.32 ± 41.91	154.09 ± 35.36	156.32 ± 35.10
Creatinine, mg/dL	0.85 ± 0.18	0.88 ± 0.19	0.85 ± 0.17	0.85 ± 0.17
Insulin. mIU/L	11.86 ± 7.24	11.78 ± 7.39	12.44 ± 8.92	13.02 ± 8.32
Na+ excretion, (mmol/24 h)	175.82 ± 77.19	181.13 ± 68.33	176.82 ± 66.43	178.37 ± 72.63
**Food groups**				
Dairy (1–2/day), %	84.2	79.2	85.1	81.5
Fruit and vegetables (2–3/day), %	81.9	76.4	83.2	79.3
Cereals (2–3 day), %	47.1	49.1	45.7	47.4
Poultry (2–3/week), %	26.7	23.1	31.7	28.4
Red and other processed meat (3–4/week), %	74.2	69.4	78.1	72.1
Nut (3–4/week), %	21.1	26.3	27.3	24.2
Red wine, 4–7/week, %	7.5	7.0	9.0	7.7

BMI: Body Mass Index; BP: Blood Pressure; MAP: Mean arterial pressure. BP was measured with 24h-ABPM (OSROM). T-paired test related samples applied, * *p* < 0.05 vs. baseline.
