# Supplementary material for: Beneficial Impact of Pork Dry-Cured Ham Consumption on Blood Pressure and Cardiometabolic Markers in Individuals with Cardiovascular Risk
Source: Nutrients. 2022 Jan 11;14(2):298. doi: 10.3390/nu14020298 (PMC8777827; doi:10.3390/nu14020298)
Supplement: Supplementary file 1 [file nutrients-14-00298-s001.zip › nutrients-1494751-supplementary-done.pdf]

nutrients

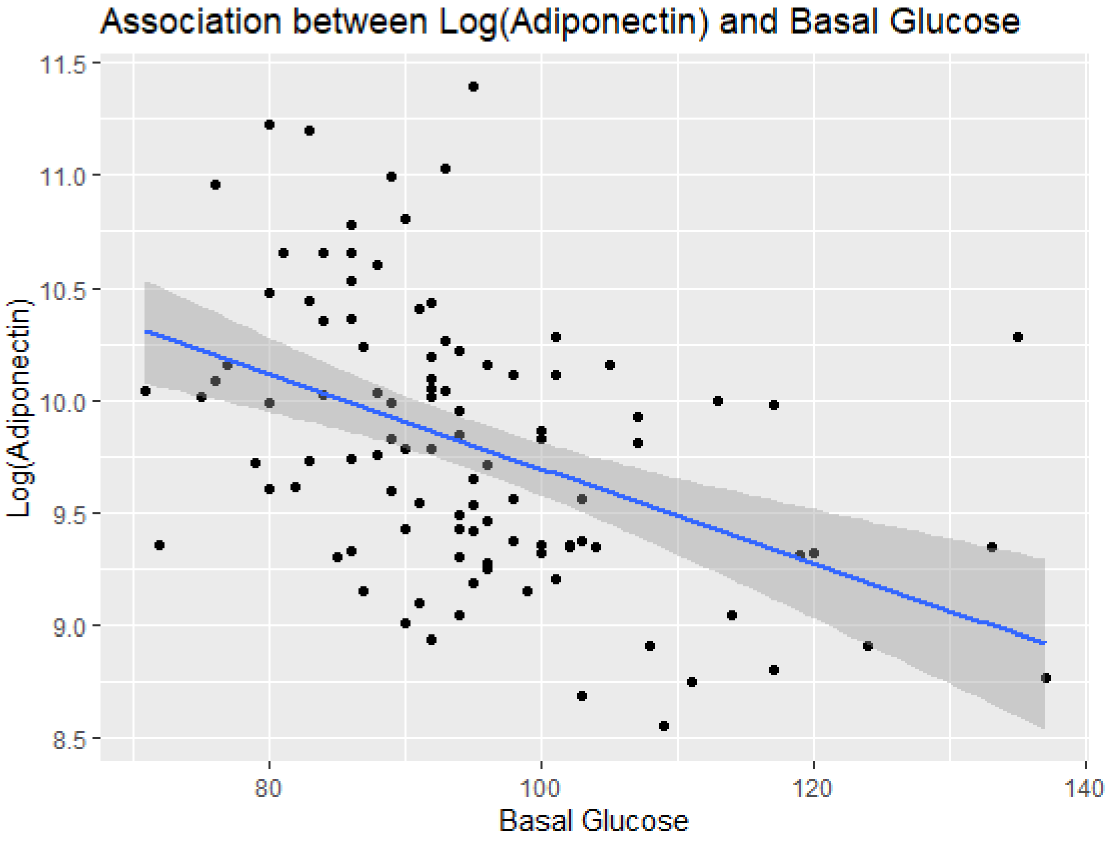


Figure S1. Linear regression and Pearson correlation between basal glucose and adiponectin at baseline status.

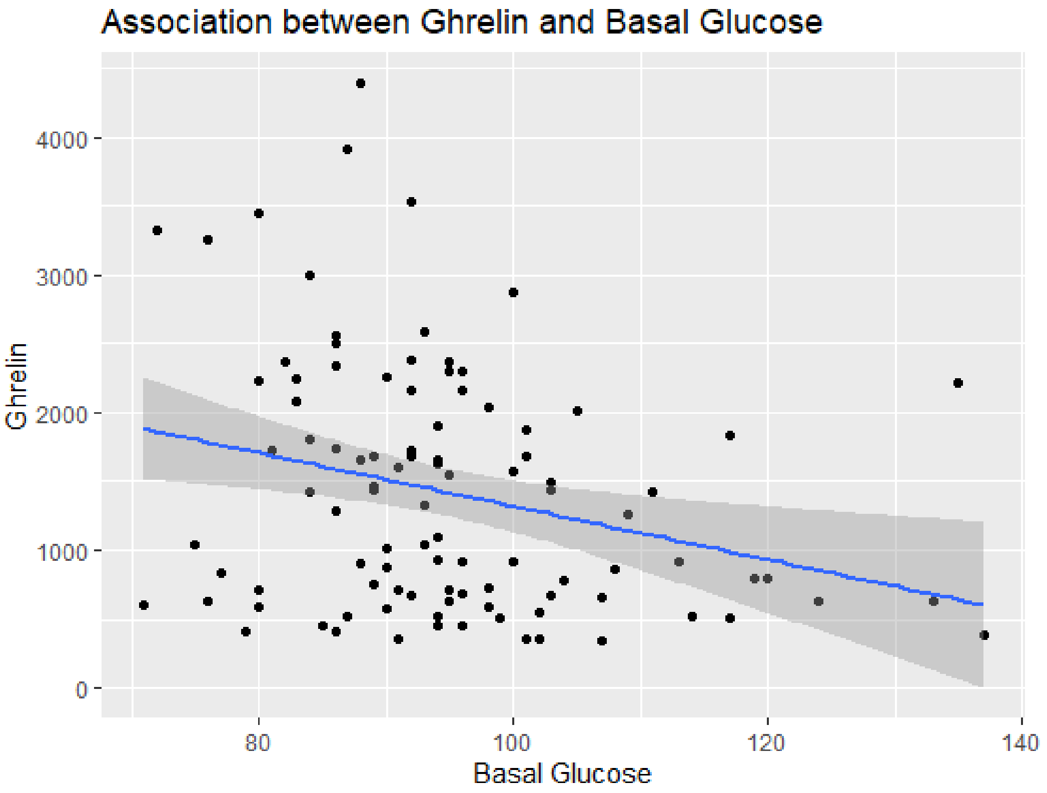


Figure S2. Linear regression and Pearson correlation between basal glucose and ghrelin at baseline status.
